# Adaptation and Validation of the Readiness for Practice Instrument for Senior Undergraduate Nursing Students in China

**DOI:** 10.1155/2023/8345744

**Published:** 2023-07-06

**Authors:** Zhenwei Dai, Jia Wang, Weiguang Ma

**Affiliations:** ^1^School of Population Medicine and Public Health, Chinese Academy of Medical Sciences and Peking Union Medical College, Beijing 100144, China; ^2^School of Nursing, Chinese Academy of Medical Sciences and Peking Union Medical College, Beijing 100144, China

## Abstract

**Background:**

The high turnover rate of new nurses is significantly impacted by the transition issue. Practice readiness has been identified as a key factor in ensuring a smooth transition, but there is currently a lack of specific instruments available in China to measure it.

**Aims:**

To translate and adapt the Casey-Fink Readiness for Practice Survey and validate its psychometric properties in senior nursing students in the Chinese cultural context.

**Methods:**

The descriptive cross-sectional study design was used. The English version of the Casey-Fink Readiness for Practice Survey was translated into Chinese according to Brislin's translation guidelines. A total of 590 senior nursing students from three nursing schools in China were surveyed with the Chinese version of the Readiness for Practice Instrument. Data analyses were performed to assess the reliability and validity of the instrument. The STROBE guidelines were used as the reporting method.

**Results:**

The validated Chinese version of the Casey-Fink Readiness for Practice Survey contained 18 items in the same four dimensions as the original English version. The internal consistency was satisfactory, with Cronbach's *α* and composite reliability values for all four dimensions above 0.70. The final confirmatory factor analysis model also demonstrated good fit indices, with the average variance extracted values for all dimensions above 0.50.

**Conclusion:**

The validated Chinese version of the Casey-Fink Readiness for Practice Survey is a reliable and effective instrument for measuring the readiness for practice among senior nursing students in China. *Implications for Nursing Management.* This study presents a valuable contribution to adapting an effective and reliable tool to assess readiness for practice among Chinese senior undergraduate nursing students. This will enable the development of specific strategies based on cultural context to optimize the readiness for practice for senior nursing students and smooth the transition of new nurses in China.

## 1. Background

Newly graduated nurses (NGNs) play a critical role in the nursing workforce as a human resource supply. The State of the World Nursing Report 2020 proposed by the World Health Organization (WHO) highlights that the nursing profession receives an influx of 1.7 million NGNs each year [1]. However, the turnover rates of NGNs were especially high, ranging from 8% to 69% in the first year and 26.2% to 57% in the second year worldwide [2, 3]. In the United States, NGNs' turnover rate was 27.6% in the first year, accounting for 32.1% of all nursing turnover in 2019 [4]. A longitudinal study in China also demonstrated that the turnover rate of NGNs was approximately 33.2% [5]. The high turnover rate further exacerbated globally the nursing shortage problem.

Previous studies have shown that a variety of factors contribute to the high turnover rate among NGNs, including their low clinical competence, coping self-efficacy, and working environment. Among these reasons, low competency is identified as one of the main reasons for their intention to leave and actual turnover [6]. Low competency usually leads to negative experiences and feelings for new nursing graduates, such as low professional confidence, high confusion and challenges, and even transition shock [7]. NGNs often express feelings of being unprepared to apply clinical knowledge and technical skills in practical settings to perform the expected professional roles and responsibilities when transitioning from an academic to a professional clinical setting. Thus, practice readiness is believed to be the key element in understanding reality/transition shock among new graduate nurses [8].

Readiness is usually described as a feeling or state of being fully prepared for the required action [9]. Practice readiness typically refers to the extent to which individuals are perceived to possess the knowledge and skills to practice autonomously [10]. Mirza et al. identified three attributes of practice readiness for newly graduated nurses, including clinical capability, cognitive capability, and professional capability [11]. Baumann et al. also identified the personal, clinical, and relational characteristics and organizational acuity as the four characteristics of practice readiness for new nurses [12]. Although different scholars may report different components of practice readiness, the maturity of nursing students, the knowledge and skills they learned in school, and their clinical practice experience during clinical practicums are believed to be the antecedents of new nurses' practice readiness [8].

Nursing students are an important resource for new nurses. Thousands of nursing students enter the nursing workforce and profession every year. In Australia, 10,000 nursing graduates enter the profession each year, and the majority of them work in acute care settings [13]. In China, 203,000 nursing graduates with advanced nursing degrees begin nursing practice each year [14]. Although nursing students do not carry the same level of roles and responsibilities as qualified clinical nurses, they still need to care for patients during clinical practicum with the professional knowledge and skills they learned in educational institutions [15]. They also face transition issues and encounter high levels of stress due to their limited clinical experience and insufficient readiness [16, 17].

Senior nursing students' readiness for practice refers to their perceptions of readiness and preparation for professional nursing roles, which are usually regarded as their ability to fulfill professional roles incorporating knowledge, behaviors, skills, and attitudes [2, 18]. Their readiness for practice largely hinges on their exposure to opportunities to practice the key dimensions of nursing during their education [19]. It has been proved that senior nursing students who possess a high level of practice readiness often experience less anxiety when transitioning to qualified nurses and are more likely to adapt to the work environment [20].

Due to the importance of practice readiness, many strategies and supports have been suggested to achieve and enhance the level of practice readiness of nursing students. The most widely employed strategies are the final clinical practicum and internship [21]. Undergraduate nursing education in China consists of a four-year program, with a clinical internship lasting over eight months in the fourth year [5]. However, nursing students are still intended to report their stressful and helpless experiences upon entering the clinical workplace [5]. Since nursing education does not always prepare nursing students for practice during the clinical practicum, they were not confident in their practice readiness [22]. Casey et al. [18] reported that only 3% of senior nursing students felt capable of independently carrying out specific skills and procedures, such as urinary catheter insertion, in an actual clinical context.

The assessment of senior nursing students' readiness for practice with a culturally appropriate assessment tool is crucial in addressing low readiness upon entering the clinical workplace. However, specific instruments to evaluate the readiness for practice of senior nursing students in China are lacking. Since many instruments have been developed in this regard, adapting and validating an appropriate instrument are more efficient solutions. There exist several assessment tools that may be used to assess the readiness for practice among senior nursing students in the Chinese cultural context. The Work Readiness Scale [23] is one of the widely used assessment tools, but its evaluation is time-consuming due to its 64 items in four dimensions. Walker improved the original Work Readiness Scale to the Work Readiness Scale for Graduate Nurses (WRS-GN), adding 9 nursing items based on the original items [24]. While Li translated and validated the WRS-GN into Chinese [25], the scale is more suitable for assessing the work readiness of new nurses rather than senior nursing students. Shahsavari developed the Perceived Professional Preparedness of Senior Nursing Students questionnaire (PPPNS, [19]), but it is more effective to evaluate nursing students' perceived preparedness to enter the clinical setting. The Casey-Fink Readiness for Practice Survey (CFRPS) [18] was developed to assess the practice readiness of senior nursing students in the United States. It was further revised and validated in the New Zealand context [26].

Therefore, this study aims to translate and validate the CFRPS and evaluate its psychometric properties in Chinese senior nursing students to provide a culturally appropriate tool for assessing their practice readiness in senior nursing students in the Chinese cultural context.

## 2. Methods

### 2.1. Parallel Translation

With the permission of the scale developer, the original English CFRPS was translated into Chinese using a parallel blind technique to facilitate an open discussion and collaboration. Two associate professors with Ph.D. degrees and experiences as visiting scholars in Britain and the United States, respectively, completed the independent translation. Then, two English teachers working in nursing colleges with master's degrees completed the independent back-translation. Subsequently, different versions of CFRPS are cross-checked and modified. The final Chinese version of the instrument was confirmed after any discrepancies were resolved through consensus among the translators.

### 2.2. Study Design and Participants

In this study, a descriptive cross-sectional design was utilized and STROBE was employed as the reporting method. Participants were recruited from three nursing schools in North China. Senior undergraduate nursing students were invited to fill out a questionnaire including demographics and the Chinese version of the CFRPS, by convenience sampling from May 2021 to May 2022. The inclusion criteria were senior nursing students who (1) were in the last year of undergraduate study; (2) complete the practicum internship; (3) were preparing for clinical practice; and (4) were willing to participate in the survey. The exclusion criteria were as follows: senior nursing students who plan to work in non-nursing fields after graduation. The minimum sample size of this study was calculated on the principle of “above ten participants per scale item” [27]. Finally, 591 senior nursing students that met the criteria above accomplished the questionnaire, and 590 samples were included in the analysis after deleting one sample questionnaire with incomplete answers and missing values.

### 2.3. Measures

In this study, two parts of the survey questionnaire were used to measure participants' practice readiness. Section A collected demographic information, including age, gender, educational level, and questions related to clinical practicum. Section B used the Casey-Fink Readiness for Practice Survey-Chinese Version (CFRPS-C) to collect data about the practice readiness of participants. The original CFRPS is a 4-point Likert scale consisting of 18 items (1 = strongly disagree to 4 = strongly agree). It has four subdomains: clinical problem-solving (7 items), learning techniques (2 items), professional identity (5 items), and trials and tribulations (4 items). Higher scores in clinical problem-solving, learning techniques, professional identity, and lower scores in trials and tribulations indicate a higher level of readiness for practice. Cronbach's *α* of the original CFRPS was 0.69 for all items and 0.80 for clinical problem-solving, 0.50 for learning techniques, 0.65 for professional identity, and 0.63 for trials and tribulations, respectively, which indicates good internal consistency reliability. Exploratory factor analysis suggested that the four-factor set of correlated subscales accounted for 48.2% of the variance across all survey items [18].

### 2.4. Statistical Analysis

A descriptive analysis was employed to examine participants' demographic characteristics. Pearson correlation analysis was employed to examine the correlation between the four subscales of CFRPS-C. A four-factor confirmatory factor analysis (CFA) with oblique rotation was performed to evaluate the reliability and validity of CFRPS-C. Due to the ordinal nature of items in CFRPS-C, mean-variance-adjusted weighted least squares (WLSMV) estimation was employed to estimate the parameters of the factor model. The structural validity of CFRPS-C was evaluated by Kaiser–Meyer–Olkin (KMO) and Bartlett's test of sphericity, and the model fit indices included *χ*^2^, d*f*, root mean square error of approximation (RMSEA), comparative fit index (CFI), Tucker–Lewis Index (TLI), and standardized root mean square residual (SRMR). The criteria of a good model fit were: *χ*^*2*^*/*d*f* < 5, RMSEA < 0.08, CFI > 0.90, TLI > 0.90, and SRMR < 0.08 [28]. The reliability and convergent validity of the model were evaluated by standardized factor loadings, Cronbach's *α*, composite reliability (CR), and average variance extracted (AVE), where CR > 0.80 and AVE > 0.50 indicate good reliability and convergent validity [29]. The discriminant validity of the model was evaluated by the bootstrap confidence interval test, where the bootstrap confidence interval not including one suggests discriminant validity [30, 31]. Statistical analysis was completed with SAS 9.4 and Mplus 8.3.

### 2.5. Ethical Considerations

Ethical approval for the survey was obtained from the Ethics Committee of the University of the Corresponding Author. All participants were informed of the purpose of this study and were free to participate in this survey. All participants gave written informed consent for their anonymized data to be used for research purposes, including publication. We also received permission from Dr. Casey (e-mail communication with Kathy Casey, 26 April 2020), the author of the CFRPS, to adapt and validate the scale in the Chinese context.

## 3. Results

### 3.1. Participant Characteristics

In this study, a total of 590 nursing students were enrolled, comprising predominantly female participants (83.1%, *n* = 490). The mean age of the participants was (21.73 ± 0.33). Notably, all of the senior nursing students had undergone an 8-month practicum in various clinical settings, including medical, surgical, gynecological, pediatric, emergency, and intensive care units.

### 3.2. Structural Validity

**Figure 1:**
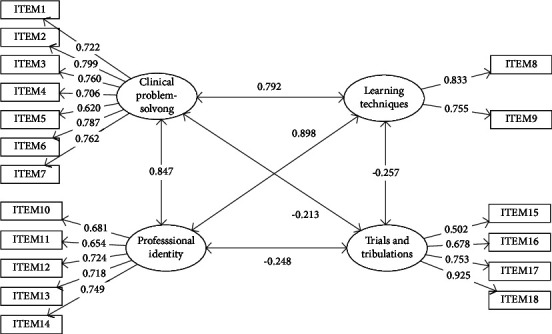
Confirmatory factor analysis of the 18-item CFRPS-C with standardized loading.

The KMO measure of CFRPS-C was 0.909, indicating enough items are predicted by each factor in the current study. The result of Bartlett's test of sphericity was statistically significant (*χ*^2^ = 4874.185, *P* < 0.001), suggesting the study data is suitable for factor analysis. The model fit indices of the confirmatory factor analysis (CFA) are illustrated in Table 1. The initial CFA model (Table 1, Model 1) with full items showed *χ*^2^/d*f* = 17.181, RMSEA = 0.166, CFI = 0.771, TLI = 0.735, and SRMR = 0.104, suggesting an unacceptable model fit. After deleting two items (“I am comfortable delegating tasks to the nursing assistant” and “I have had opportunities to practice skills and procedures more than once” in Trials and Tribulations) with low standardized factor loadings (<0.5), the CFA model (Table 1, Model 2) showed improved fit indices: *χ*^2^/d*f* = 7.329, RMSEA = 0.104, CFI = 0.922, TLI = 0.907, and SRMR = 0.060. To obtain the optimal and most parsimonious model, modification indices were evaluated and the correlations between error terms were added to further improve the model fit. The final model fit indices (Table 1, Model 3) were *χ*^2^/d*f* = 4.562, RMSEA = 0.078, CFI = 0.958, TLI = 0.948, and SRMR = 0.046, indicating the final CFA model showed good structural validity.  Figure 1 displays the standardized loadings for the final 18-item CFA model of CFRPS-C.

### 3.3. Reliability and Convergent Validity

All four dimensions of the 18-item CFRPS-C showed statistically significant and satisfactory reliability and convergent validity, as evidenced by the standardized factor loadings above 0.5 and significant (see Figure 1 and Table 2), Cronbach's *α* and composite reliability values (CR) above 0.7 (see Table 2). The average variance extracted values (AVE) of the four dimensions were all equal to or above the recommended value of 0.5 (see Table 2).

### 3.4. Discriminate Validity

The absolute values of the correlation coefficients between the four dimensions of the 18-item CPRS-C ranged from 0.213 to 0.898 (see Table 3). The confidence interval of correlations derived from 5000 bootstrap samplings all did not include 1, suggesting acceptable discriminate validity of the 18-item CFRPS-C [30, 31].

## 4. Discussion

Clinical internships are a crucial period for nursing students to experience clinical nursing and develop a professional identity, enabling them to transit smoothly into professional clinical nurses [32]. Yet we have no specific instruments to evaluate the practice readiness of senior nursing students during this critical period in China. Therefore, the primary objective of this study was to translate and adapt the Casey-Fink Readiness for Practice Survey (CFRPS) into the Chinese version through a parallel translation process.

The Chinese version of the CFRPS, which is an 18-item scale, has been validated through a parallel translation process. The results of the confirmatory factor analysis (CFA) showed that all the standardized factor loadings of the final 18-item CFRPS-C was statistically significant and above the recommended value of 0.5, indicating good commonalities of items [33]. Furthermore, the values of Cronbach's *α* and CR for the four dimensions were above 0.7, and the average variance extracted (AVE) of the dimensions was above 0.5. This suggests that the Chinese version of CFRPS has acceptable reliability and convergent validity [28]. In addition, the discriminate validity of the CFA model was acceptable according to the correlation coefficients between the four dimensions [30, 31]. Therefore, the findings indicated that the Chinese version of CFRPS is reliable and valid for the evaluation of readiness for practice in Chinese senior nursing students.

In the original CFRPS, Casey identified four factors closely related to the readiness for practice, including clinical problem-solving, learning techniques, professional identity, and trials and tribulations. Previous studies also focused on these domains singly or combined to reflect the practice readiness of nursing students or new nurses [8]. In our study, we found the final Chinese version of CFRPS has 18 items in the same dimensions as the original CFRPS. What's more, all the items in the dimensions of clinical problem-solving skills, learning techniques, and professional identity have completely remained, indicating that these factors can also assess the practice readiness of Chinese senior nursing students. But in this study, two items in the “Trials and Tribulations” dimension were deleted from the original CFRPS, namely, “I am comfortable delegating tasks to the nursing assistant” and “I have had opportunities to practice skills and procedures more than once” due to their poor interpret-ability to the whole Chinese version of the CFRPS. In addition, these items are not consistent with the Chinese nursing education background. During the 8-month clinical internships, the roles and responsibilities of Chinese senior nursing students are almost the same as those of clinical nurses. They all have many opportunities to practice nursing skills and procedures [5]. But they have to practice under the supervision of experienced clinical preceptors without nursing licenses [34]. Thus, the deletion of these two items also implied the importance of the scale cultural adjustment.

## 5. Strengths and Limitations

From a methodological perspective, this study has notable strengths. First, to ensure the accuracy of the translation and adaptation process, four experts who are knowledgeable about undergraduate nursing education issues in China and possess good English language skills were involved. Second, the use of CFA in the validation process enabled the assessment of construct validity. Finally, with a sample size of 590 Chinese senior nursing students who fully completed the questionnaires, this study had a sufficiently large sample for psychometric testing.

This study has several limitations. First, the convenience sampling method used in this study may limit the generalization of the findings. In addition, the study only included senior nursing students from one region in China, which may not be representative of senior nursing students in other regions. Further studies that include participants from various regions are necessary to confirm the validity of the Chinese version of CFRPS. Another limitation of the study is that the CFRPS is a self-report instrument, which may be influenced by social desirability bias. Participants may give a subjective or socially appropriate answer that does not accurately reflect their actual level of practice readiness. Future studies that use a combination of qualitative and quantitative methods may provide more comprehensive and accurate data. Besides, the survey was conducted from May 2021 to May 2022. The clinical experience of senior nursing students may be limited due to the online clinical practicum during the COVID-19 pandemic. Thus, the result obtained in this study is difficult to work as the norm for practice readiness of Chinese senior nursing students to compare with other studies.

## 6. Conclusion

The 18-item CFRPS-C demonstrated good construct validity and provides stronger psychometric support for the original CFRPS. Thus, the CFRPS-C is a useful tool to assess practice readiness among Chinese senior nursing students. Its brevity also enhances its convenience and practicality in clinical settings.

## 7. Implications for Nursing Management

New Nurses are the main supplier to the nursing workforce, and the transition issue contributes a lot to their retention rate. Nursing management should recognize the importance of practice readiness in the clinical practicum for senior nursing students, as it is a critical factor in their transition to professional clinical nurses and can affect their high turnover rate. Yet there is a lack of specific instruments to evaluate the readiness for practice in China. This study's adaptation and validation of the Readiness for Practice Instrument for Chinese senior undergraduate nursing students provide a valuable tool for evaluating practice readiness in China. Data from surveys with this scale in China may enrich and deepen the understanding of readiness for practice. Culturally specific strategies should be developed based on different cultures to optimize the readiness for practice and promote retention of the nursing profession.

## Figures and Tables

**Table 1:** Model fit of the confirmatory factor analysis.

Model	*χ* ^*2*^	d*f*	*χ* ^*2*^ * /*d*f*	RMSEA	CFI	TLI	SRMR
Model 1	2817.675	164	17.181	0.166	0.771	0.735	0.104
Model 2	945.510	129	7.329	0.104	0.922	0.907	0.060
Model 3	565.700	124	4.562	0.078	0.958	0.948	0.046

**Table 2:** Reliability and convergent validity of the 18-item CFRPS-C.

Dimension	Indicator	STD	SE	*Z*	*P*	Cronbach's *α*	CR	AVE
Clinical problem-solving	Feel confident communicating with physicians	0.722	0.022	32.536	<0.001	0.828	0.893	0.546
	I am confident in my ability to problem solve	0.799	0.017	46.505	<0.001			
	I use current evidence to make clinical decisions	0.760	0.018	42.834	<0.001			
	I am comfortable communicating and coordinating care with interdisciplinary team members	0.706	0.022	32.439	<0.001			
	I feel comfortable knowing what to do for a dying patient	0.620	0.023	27.022	<0.001			
	I feel comfortable taking action to solve problems	0.787	0.018	43.402	<0.001			
	I feel confident identifying actual or potential safety risks to my patients	0.762	0.018	42.243	<0.001			

Learning techniques	Simulations have helped me feel prepared for clinical practice	0.833	0.018	46.488	<0.001	0.701	0.774	0.632
	Writing reflective journals/logs provided insights into my own clinical decision-making skills	0.755	0.019	38.86	<0.001			

Professional identity	Feel comfortable communicating with patients and their families	0.681	0.020	33.957	<0.001	0.781	0.832	0.500
	My clinical instructor provided feedback about my readiness to assume an RN role	0.654	0.022	29.165	<0.001			
	I am comfortable asking for help	0.724	0.020	37.088	<0.001			
	I am satisfied with choosing nursing as a career	0.718	0.021	34.68	<0.001			
	I feel ready for the professional nursing role	0.749	0.020	38.216	<0.001			

Trials and Tribulations	I have difficulty documenting care in the electronic medical record	0.502	0.038	13.338	<0.001	0.778	0.814	0.534
	I have difficulty prioritizing patient care needs	0.678	0.030	22.709	<0.001			
	I feel overwhelmed by ethical issues in my patient care responsibilities	0.753	0.028	27.338	<0.001			
	I have difficulty recognizing a significant change in my patient's condition	0.925	0.022	41.464	<0.001			

*Note.* STD, standardized factor loading; SE, standard error.

**Table 3:** Pearson correlation and discriminate validity of the 18-item CFRPS-C.

	D1	D2	D3	D4
D1	1			
D2	0.792 (0.691∼0.892)	1		
D3	0.847 (0.801∼0.891)	0.898 (0.801∼0.994)	1	
D4	−0.213 (−0.361∼−0.064)	−0.257(−0.412∼−0.102)	−0.248(−0.405∼−0.091)	1

*Note.* D1 = clinical problem-solving; D2 = learning techniques; D3 = professional identity; D4 = trials and tribulations.

## Data Availability

The datasets generated and analyzed during the current study are not publicly available to ensure data confidentiality.

## Additional Points

*What Does This Paper Contribute To the Wider Global Clinical Community?* (1) It adapts and validates a reliable and valid instrument to measure the readiness for practice among Chinese senior nursing students. (2) It provides increased psychometric support for the original Casey-Fink Readiness for Practice Survey. (3) It makes it possible to enrich the understanding of readiness for the practice among different cultures with the same instrument, to optimize the readiness, and help retention of the nursing profession.
